# Best hits of 11110110111: model-free selection and parameter-free sensitivity calculation of spaced seeds

**DOI:** 10.1186/s13015-017-0092-1

**Published:** 2017-02-14

**Authors:** Laurent Noé

**Affiliations:** CRIStAL (UMR 9189 Lille University/CNRS)-Inria Lille, Bat M3 ext, Université Lille 1, 59655 Villeneuve d’Ascq, France

**Keywords:** Spaced seeds, Dominant seeds, Bernoulli, Hit Integration, Heaviside, Dirac, Counting semi-ring, Polynomial form, DFA

## Abstract

**Background:**

*Spaced seeds*, also named *gapped q-grams, gapped k-mers, spaced q-grams*, have been proven to be more sensitive than contiguous seeds (*contiguous q-grams, contiguous k-mers*) in nucleic and amino-acid sequences analysis. Initially proposed to detect sequence similarities and to anchor sequence alignments, spaced seeds have more recently been applied in several *alignment-free* related methods. Unfortunately, spaced seeds need to be initially designed. This task is known to be time-consuming due to the number of spaced seed candidates. Moreover, it can be altered by a set of *arbitrary chosen* parameters from the probabilistic alignment models used. In this general context, *Dominant seeds* have been introduced by Mak and Benson (Bioinformatics 25:302–308, 2009) on the Bernoulli model, in order to reduce the number of spaced seed candidates that are further processed in a *parameter-free* calculation of the sensitivity.

**Results:**

We expand the scope of work of Mak and Benson on single and multiple seeds by considering the Hit Integration model of Chung and Park (BMC Bioinform 11:31, 2010), demonstrate that the same dominance definition can be applied, and that a parameter-free study can be performed without any significant additional cost. We also consider two new discrete models, namely the Heaviside and the Dirac models, where lossless seeds can be integrated. From a theoretical standpoint, we establish a generic framework on all the proposed models, by applying a *counting semi-ring* to quickly compute large polynomial coefficients needed by the *dominance* filter. From a practical standpoint, we confirm that *dominant seeds* reduce the set of, either single seeds to thoroughly analyse, or multiple seeds to store. Moreover, in http://bioinfo.cristal.univ-lille.fr/yass/iedera_dominance, we provide a full list of spaced seeds computed on the four aforementioned models, with one (continuous) parameter left free for each model, and with several (discrete) alignment lengths.

## Background

*Optimized spaced seeds*, or *best gapped q-grams*, have independently been proposed in PatternHunter [3] and by Burkhardt and Karkkainen [4]. The primary objective was either to improve the sensitivity of the heuristic but efficient *hit and extend* BLAST-like strategy (without using the *neighborhood word principle* 1), or to increase the selectivity for lossless filters on alignments of size $\ell$ under a given Hamming distance of *k*.

Several extensions of the spaced seed model have then been proposed on the two aforementioned problems: vector seeds [5], one gapped *q*-grams [6] or indel seeds [7, 8], neighbor seeds [9, 10], transition seeds  [11–15], multiple seeds [16–19], adaptive seeds [20] and related work on the associated indexes  [21–26], just to mention a few.

Unfortunately, spaced seeds are known to produce hard problems, both on the seed sensitivity computation [27] or the lossless computation [28], and moreover on the seed design [29]. But the choice of the right seed pattern has a significant impact on genomic sequence comparison [3, 12, 16, 20, 30–38], on oligonucleotide design [39–44], as well as on amino acid sequence comparison [45–53]; this has led to several effective methods to (possibly greedily) select spaced seeds [54–61] with elaborated alignment models and their associated algorithms [62–70].

Another less frequently mentioned problem is that the seed design is mostly performed on a *fixed and already fully parameterized* alignment model (for example, a *Bernoulli* model where the *probability of a match* *p* is set to 0.7). There is not so much choice for the optimal seed, when, for example, the scoring system is changed, and thus the expected distribution of alignments.

We note that several recent works mention the use of spaced seeds in *alignment-free* methods [71–73] with applications in phylogenetic distance estimation [74], metagenomic classification [75, 76], just to cite a few.

Finally, we also noticed that several recent studies use the *overlap complexity* [54, 56, 57, 77–79] which is closely linked to the *variance* of the number of spaced-word matches [80] and is known to provide an upper/lower bound for the expectation of the length preceding the first seed hit [27, 66, 81]. We mention here that a similar *parameter-free* approach could also be applied for the *variance induced* selection of seeds, but an interesting question remains in that case: to find a *dominance equivalent* criterion associated with the selection of candidate seeds.

The paper is organized as follows. We start with an introduction to the *spaced seed model* and its associated *sensitivity* or *lossless aspect*, and show how *semi-rings* on DFA can help determining such features. Section “Semi-rings and number of alignments” restricts the description to *counting semi-rings* that are applied on a specific DFA to perform an efficient dynamic programming algorithm on a set of counters. This is a prerequisite for the two next sections that present respectively *continuous models* and *discrete models*. Section “Continuous models” is divided into two parts : the first one outlines the *polynomial form of the sensitivity* proposed by [1] to compute the sensitivity on the *Bernoulli model* together with the associated *dominance principle*, whereas the second one extends this *polynomial form* to the *Hit Integration model* of [2], and explains why the dominance principle remains valid. Section “Discrete models” describes two new *Dirac* and *Heaviside* models, and shows how *lossless seeds* can be integrated into them. Then, we report our experimental analysis on all the aforementioned models, display and explain several optimal seed Pareto plots for the restricted case of one single seed, and links to a wide range of compiled results for multiple seeds. The last section brings the discussion to the asymptotic problem, and to several finite extensions.

## Spaced seeds and seed sensitivity

We suppose here that strings are indexed starting from position number 1. For a given string *u*, we will use the following notation: *u*[*i*] gives the *i*-th symbol of *u*, |*u*| is the length of *u*, and $|u|_a$ is the number of symbol letters *a* that *u* contains.

Nucleotide sequence alignments without *indels* can be represented as a succession of *match* or *mismatch* symbols, and thus represented as a string *x* over a binary alphabet $\{\texttt {0},\texttt {1}\}$.

A spaced seed can be represented as a string $\pi$ over a binary alphabet $\{\text {0},\text {1}\}$ but with a different meaning for each of the two symbols: $\text {1}$ indicates a position on the seed $\pi$ where a single *match* must occur in the alignment *x* (it is thus called a *must match* symbol), whereas $\text {0}$ indicates a position where a single *match* or a single *mismatch* is allowed (it is thus called a *don’t-care* symbol).

The *weight* of a seed $\pi$ (denoted by *w* or $w_\pi$) is defined as the number of *must match* symbols ($w_\pi = |\pi |_1$): the weight is frequently set constant or with a minimal value, because it is related to the *selectivity* of the seed. The *span* or *length* of a seed $\pi$ (denoted by $s_\pi$) is its full length ($s_\pi = |\pi |$). We will also frequently use $\ell$ for the length of the alignment ($\ell =|x|$).

The spaced seed $\pi$ *hits* at position *i* of the alignment *x* where $i \in \big [1\ldots\,|x|-|\pi |+1\big ] = \big [1\ldots\,\ell -s_\pi +1\big ]$ iff

$$\begin{aligned} \forall j \in \big [1\ldots\,s_\pi \big ] \qquad \pi [j] = \text {1}\implies x[j+i-1] = \texttt {1} \end{aligned}$$

For example, the seed $\pi = \text {1101}$ hits the alignment $x = \texttt {111010101111}$ twice, at positions 2 and 9. 
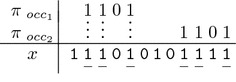


Naturally, the shape of the seed, i.e.  possible placement of a set of *don’t-care* symbols between any consecutive pair of the *w* *must match* symbols, plays a significant role and must be carefully controlled. Requiring *at least one hit* for a seed, on an alignment *x*, is the most common (but not unique) way to select a *good seed*.

However, depending on the context and the problem being solved, even measuring this simple feature can easily take one of the two (previously briefly mentioned) forms:

- When considering that any alignment *x* is of given length $\ell$, and each symbol is generated by a Bernoulli model (so there is no restriction on the number of match or mismatch symbols an alignment must contain, but with some configurations more probable than others), the problem is to select a *good seed* (respectively the *best seed*) as the one that has a *high probability* (respectively the *best probability*) to hit at least once.
- When considering that any alignment *x* is of given length $\ell$, and contains at most *k* mismatch symbols, a classical requirement for a *good seed* is to guarantee that *all the possible alignments*, obtained by any placements of *k* mismatch symbols on the $\ell$ alignment symbols, will *all* be detected by at least one seed hit each: when this distinctive feature occurs, the seed is considered *lossless* or $(\ell ,k)$ *-lossless*.

**Fig. 1:**
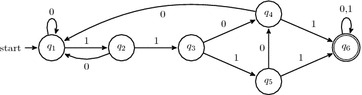
Spaced seed DFA. We represent the *at least one hit* DFA for the spaced seed $\pi = \text {1101}$. This automaton recognizes any alignment sequence with at least one occurrence of $\texttt {1101}$ or $\texttt {1111}$

The two problems can be solved by first considering the language recognized by the seed $\pi$, in this context the *at least one hit*  regular language, and its associated DFA. As an illustration, Fig.  1 displays the *at least one hit*  DFA for the spaced seed $\text {1101}$: this automaton recognizes the associated regular language $\{\texttt {0},\texttt {1}\}^{*} ( \texttt {1101} | \texttt {1111}) \{\texttt {0},\texttt {1}\}^{*}$, or less formally, any binary alignment sequence *x* that has *at least one* occurrence of $\texttt {1101}$ or $\texttt {1111}$ as a factor.

The second step consists in computing, by using a simple dynamic programming (DP) procedure set for any states of the DFA and for each step $i \in \big [1\ldots\,\ell \big ]$,

- Either, the probability to reach any of the automaton states.
- Otherwise, the minimal number of mismatch symbols 0 that have been crossed to reach any state.

For example, considering the probability problem (a) on a Bernoulli model where a *match* has a probability *p* set to 0.7, we show it can be computed—by first *“replacing”*, on the automaton of Fig. 1, the transition symbols 0 and 1 by their respective probabilities 0.3 and 0.7—then, on each step *i*, it is possible to compute the probability $\mathscr {P}(i,q)$ to reach each of the states *q* by applying a recursive formula that uses the probability to be at any of its preceding states on step $i-1$. For the automaton of Fig.  1, this gives  on step $i=4$, the probability to reach the final state $q_6$ can be computed to $\mathcal {P}(4,q_6)= 0.343$ ( $0.7^3$ ), as a logical (and first non-null) probability for the seed $\pi = \text {1101}$ to detect alignments of length $\ell =4$—on step $i=5$, the probability to reach $q_6$ can be computed to $\mathcal {P}(5,q_6) = 0.51793$ ($0.7^3 \times (1 + 0.3 + 0.7 \times 0.3))$ to detect alignments of length $\ell =5$ .

Another example, considering now the lossless property (b) for the spaced seed $\pi = \text {1101}$: we can show that this seed is lossless for one single mismatch, when $\ell \ge 6$ (but computational details are left to the reader, after a remark on *tropical semi-rings* in the next paragraph): the seed is thus $(\ell =6,k=1)$-lossless ; however, this seed is not $(\ell =5,k=1)$-lossless, since reading the consistent sequence $\texttt {10111}$ leads to a non-final state.

Finally, we simply mention that this second computational step involves the implicit use of *semi-rings*,

- Either *probability semi-rings*: $(E = \mathbb {R}_{0 \le r \le 1},\; \oplus = +,\; \otimes = \;\times \;,\; 0_{\oplus ,\epsilon _\otimes } = 0,\; 1_{\otimes } = 1)$ ; the final state(s) of the DFA give(s) the probability of having *at least one hit* after $\ell$ steps of the DP algorithm,
- Otherwise *tropical semi-rings*: $(E = \mathbb {R}_{\ge 0},\; \oplus = min,\; \otimes = +\;\; 0_{\oplus ,\epsilon _\otimes } = \infty ,\; 1_{\otimes } = 0)$. The seed is $(\ell ,k)$ *-lossless* iff all the non-final states of the DFA have a minimal number of mismatches that is strictly greater than *k*, after $\ell$ steps of the DP algorithm.^2^

## Semi-rings and number of alignments

Semi-rings are a flexible and powerful tool, employed for example to compute probabilities, scores, distances, counts (to name a few) in a generic dynamic programming framework [82, 83]. The first problem involved, mentioned at the end of the previous section, is the right choice of the semi-ring, adapted to the question being addressed. Sometimes, selecting an alternative semi-ring to *count elements*, may turn out to be a flexible choice that solves more involved problems (for example *computing probabilities* is one of them, and will be described in next section).

**Fig. 2:**
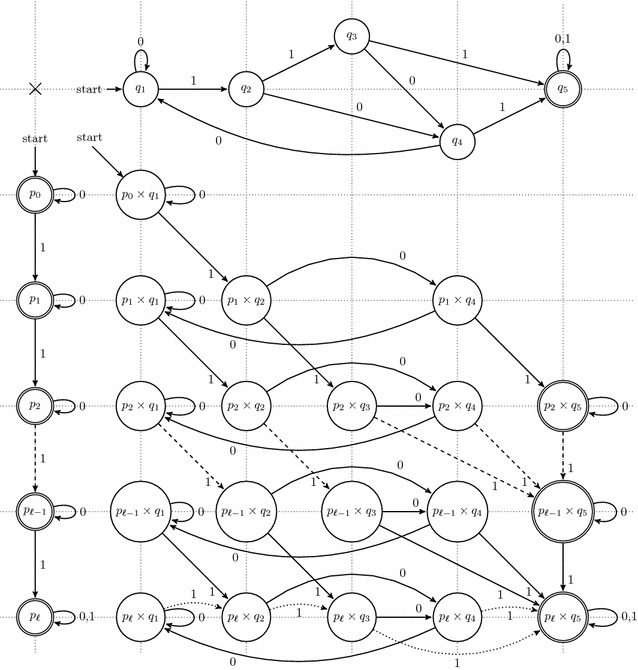
DFA intersection product. We represent the resulting intersection product of the *at least one hit* DFA for the seed $\pi = \text {101}$ (*top horizontal* automaton), with the $\text {1}$-counting DFA (*left vertical* automaton). The* dashed* transitions represent ellipsis in the construction between $m=2$ and $m=\ell -1$, while the* dotted* transitions at the bottom of the resulting automaton make it complete

Counting semi-rings [84] are adapted for this task: when applied on the *right language* and its *right automaton*, they can report the number of alignments $c_{\pi ,m}$ that are at the same time detected by the seed $\pi$ while having *m* matches out of $\ell$ alignment symbols. The main idea that enables the computation of these $c_{\pi ,m}$ counting coefficients (illustrated on Fig.  2 as the intersection product) is first to intersect the language recognized by the seed $\pi$ (the *at least one hit* language of $\pi$) with the classes of alignments that have exactly *m* matches: the automaton associated with all of these classes of alignments with *m* matches has a very simple linear form with $\ell +1$ states, where several distinct final states are defined according to all the possible values of $m \in [0\ldots\,\ell ]$. Finally, since the intersection of two regular languages is regular [Theorem 4.8 of the timeless 85], it can thus be represented by a conventional DFA, while keeping the feature of having several distinct final states.

As an illustration, Fig.  2 displays the *at least one hit* DFA for the spaced seed $\text {101}$ (on the top), the linear $\text {1}$-counting DFA (on the vertical left part) to isolate alignments with exactly *m* matches, and finally their intersection product, that represent the intersecting language as a new DFA (itself obtained by crossing *synchronously* the two previous DFAs). Note that each of the final states $p_m \times q_5$ (for $m < \ell$) of the resulting DFA is reached by alignment sequences with exactly *m* matches that are also detected by the seed $\text {101}$ (unless for the last state $p_l \times q_5$, where $\ge \ell$ matches may have been detected).

Then, starting with the empty word (counted once from the initial state $p_0 \times q_1$), it is then possible to count the number of words of size one (two words 0 and 1 on a binary alphabet) by following transitions from the initial state to $p_0 \times q_1$ and $p_1 \times q_2$, respectively; from the (two) states already reached, it is then possible to count words of size two (four words on a binary alphabet), and so on, while keeping, for each DFA state $p_m \times q_j$ and on each step *i*, a *single count* record, which represents the size of the subset of the partition of the $2^i$ words that reach $p_m \times q_j$.

Note that, for a seed $\pi$ of weight $w_\pi$ and span $s_\pi$ (thus with $s_\pi -w_\pi$ *don’t-care* symbols), the *at least one hit* automaton size is in $\mathcal {O}(w_\pi 2^{s_\pi -w_\pi })$, so the intersection with the classes of alignments that have *m* matches out of $\ell$ leads to a full size in $\mathcal {O}(\ell w_\pi 2^{s_\pi -w_\pi })$: the computational complexity of the algorithm can thus be estimated in $\mathcal {O}(\ell ^2 w_\pi 2^{s_\pi -w_\pi })$ in time and $\mathcal {O}(\ell w_\pi 2^{s_\pi -w_\pi })$ in space. As shown by [1], it can be processed incrementally for all the alignment lengths up to $\ell$, with the only restriction that the numbers of alignments per state ($\le 2^\ell$) fit inside an integer word (64 or 128bits).

We first mention that a *breadth-first* construction of the intersection product can be used to limit the *depth* of the reached states to $\ell$. We have already noticed that several authors have performed equivalent tasks with a matrix for the full automaton [86], or with a vector for each automaton state [1], probably because contiguous memory performance is better. An advantage of such lazy automaton product evaluation may be that, besides the fact that it is a *generic* automaton product, we avoid *sparse data-structures* combined with *many non-reachable* states (for example, $p_{\ell -1} \times q_1$ and $p_{\ell } \times q_1$ will never be reached on any sequences of size $\ell > 2$: since two *mismatches* are needed to reach them, then $p_m$ must always have its associated number of *matches* $m \le \ell -2$).

We finally mention that a similar method was used in [87] to compute correlation coefficients between the seed *number of hits* or the seed *coverage*, and the *true* alignment Hamming distance.^3^

In the following sections, we will use the (*m*-matches counting) $c_{\pi ,m}$ coefficients to compute, either probabilities on continuous models, or frequencies on discrete models.

## Continuous models

### Bernoulli polynomial form and dominance between seeds

Once the $c_{\pi ,m}$ coefficients (the number of alignments of length $\ell$ with *m* matches that are detected by the seed $\pi$) are determined, the probability to hit an alignment of length $\ell$ under a Bernoulli model (where the probability of having a match is *p*) can be directly computed as a polynomial over *p* of degree at most $\ell$:

1 $$\begin{aligned}Pr_\pi (p,\ell ) &= c_{\pi ,0} \, p^0 (1-p)^\ell + c_{\pi ,1}\, p^1 (1-p)^{\ell -1} +\cdots \nonumber \\& \quad \cdots + c_{\pi ,\ell -1}\, p^{\ell -1} (1-p)^{1} + c_{\pi ,\ell }\, p^\ell (1-p)^0 \end{aligned}$$

The expression (1) was first proposed by [1] for spaced seeds, noticing that each alignment with *m* match symbols and $\ell -m$ mismatch symbols, *“no matter how arranged”*, has the same probability $p^m (1-p)^{\ell -m}$ to occur. The coefficient $c_{\pi ,m}$ then gives the number of such (obviously independent) alignments that are detected by the seed $\pi$. This leads, for all the possible number of match/mismatch symbols in an alignment of length $\ell$, to the expression (1) of the sensitivity for $\pi$. At first sight, we would conclude that this formula might be numerically unstable without any adapted computation, due to large $c_{\pi ,m}$ coefficients, opposed to rather small $p^m (1-p)^{\ell -m}$ probability values. But we will see that this expression (1) is not so frequently evaluated, and when it is, requires more involved tools than a classical numerical computation.

Mark and Benson [1] also include in their paper an elegant and simple *partial order* named dominance between seeds: suppose that two spaced seeds $\pi _a$ and $\pi _b$ have to be compared according to their respective $c_{\pi _a,m}$ and $c_{\pi _b,m}$ coefficients: now, assume that, $\forall m \in [0\ldots\,\ell ] \quad c_{\pi _a,m} \ge c_{\pi _b,m}$ (with at least a single difference on at least one of the coefficients), then we can conclude that $\pi _a$ *dominates* $\pi _b$, and thus that $\pi _b$ can be discarded from the possible set of optimal seeds. Indeed, the sensitivity, defined by the formula (1) as a sum of *same positive* terms $p^m (1-p)^{\ell -m}$ , each term being respectively multiplied by a *seed-dependent positive* coefficient $c_{\pi ,m}$, guarantee that the sensitivity of $\pi _b$ will never be better than the sensitivity of $\pi _a$, whatever parameter $p \in [0,1]$ is chosen.

In practice, from the initial set of all the possible seeds of given weight *w* and maximal span *s*, several seeds can be discarded using this dominance principle, reducing the initial set to a small subset of candidate seeds to optimality. But this *dominance principle* is a *partial order* between seeds: this signifies that some seeds *cannot* be compared.

**Table 1:**
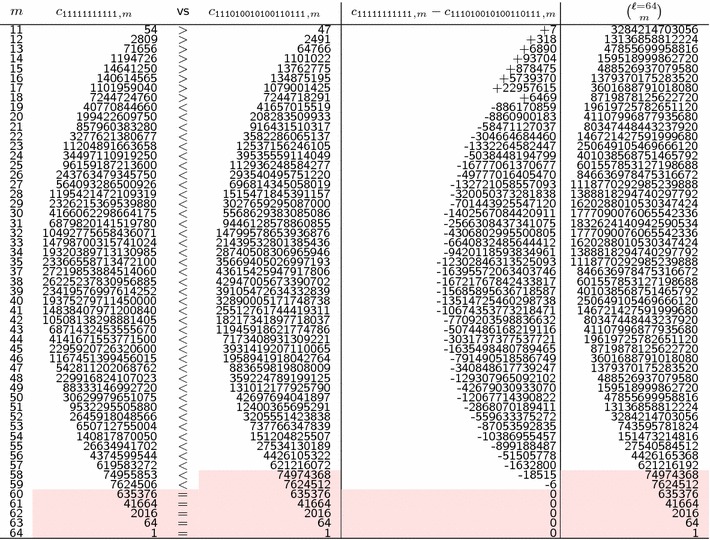
Polynomial coefficients

Number $c_{\pi ,m}$ of alignments of length $\ell =64$ with exactly *m* matches that are hit, by the contiguous seed (first column), by the Patternhunter I spaced seed (second column), and their respective difference (third column). The fourth column indicates the maximal number of alignments of length $\ell =64$ with exactly *m* matches that could have been detected: when equality occurs with the first or the second column, the seed is then considered to be *lossless*: when this occurs, the background of the cell is pink

As an illustration, Table 1 lists the $c_{\pi ,m}$ coefficients of two single seeds, the contiguous seed (11111111111), and the Patternhunter I spaced seed (111010010100110111), for the alignment length $\ell =64$. Note that comparing only the pairs of coefficients $c_{\mathtt{11111111111},m}$ and $c_{\mathtt{111010010100110111},m}$ does not help in choosing/discarding any of the two seeds by the dominance principle, since $c_{\mathtt{11111111111},m} > c_{\mathtt{111010010100110111},m}$ when $m \le 18$, or $c_{\mathtt{11111111111},m} \le c_{\mathtt{111010010100110111},m}$ otherwise (with a strict inequality when $m \le 59$). Actually, both seeds are included in the set of the dominant seeds of weight $w=11$ found on alignments of length $\ell =64$, as mentioned by [1], and verified in our experiments.

Surprisingly, according to the experiments of [1], very few single seeds are *overall dominant* in the class of seeds of same weight *w* and fixed or restricted span *s* (e.g. $s \le 2\times w$) : this *dominance* criterion was thus used as a filter for the pre-selection of optimal seeds. In the section *“Experiments”* , we show that the dominance selection also scales reasonably well for selecting multiple seeds candidates.

### Hit Integration and its associated polynomial form

*Hit Integration (HI)* for a given seed $\pi$ was proposed by [2] as $\frac{\int _{p_a}^{p_b} Pr_\pi (p,\ell ) \, dp}{p_b-p_a}$ for a given interval $[p_a,p_b]$ (with $0 \le p_a < p_b \le 1$), where $Pr_\pi (p,\ell )$ is the probability for the seed $\pi$ to hit an alignment of length $\ell$ generated by a Bernoulli model of parameter *p*, as mentioned at the beginning of the previous part.

The main idea behind this integral formula is that, to cope with a “once set” and “single” *p* value that gives higher probabilities to alignments with percent identities close to *p*, a given interval $[p_a,p_b]$ is more suitable. In terms of the generative process, $\frac{\int _{p_a}^{p_b} Pr_\pi (p,\ell ) \, dp}{p_b-p_a}$ can be *interpreted* as choosing uniformly a value for the Bernoulli parameter *p* in the range $[p_a,p_b]$, each time and once per alignment sequence, before running the Bernoulli model to generate this full alignment sequence *x* of length $\ell$.

**Fig. 3:**
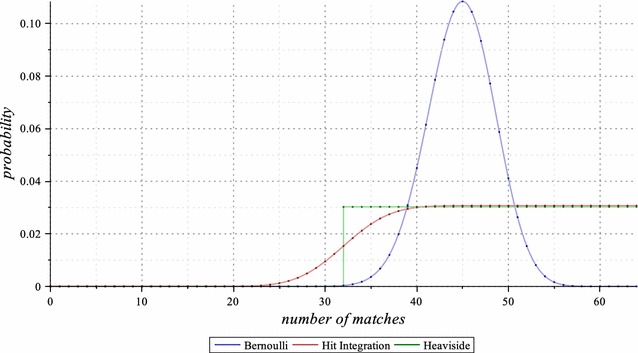
Bernoulli, Hit Integration, and Heaviside models. The Bernoulli (for $p = 0.7$), the $\int _{0.5}^{1.0}$ Hit Integration, and the $\sum _{\frac{1}{2}}^{1}$ Heaviside probability mass functions of the number of matches, on alignments of length $\ell =64$. Highlighted dots indicate the weights given for each alignment class with a given number of matches *m* out of $\ell$ alignment symbols, under each of the three models. Note that, since the sum of the weights is always 1 for any model, and since the class of alignments with exactly $m=32$ matches out of $\ell =64$ is fully included in $\sum _{\frac{1}{2}}^{1}$ Heaviside model but only half-included in $\int _{0.5}^{1.0}$ Hit Integration model, there is a thin difference between the two resulting lines

An illustration of the full probability mass function for the *Hit Integration* compared with the *Bernoulli* and the *Heaviside* distributions (the latter is defined in the next section) is given in Fig. 3 for alignments of length $\ell =64$.

Chung and Park [2] pointed out that designed spaced seeds were of different shapes, and that several seeds obtained on $[p_a=0, p_b=1]$ or $[p_a=0.5, p_b=1]$ were *in practice* better (compared with three other criteria tested in their paper). We also noticed that the method of [2] was modeled on the [27] recursive decomposition, and is based on a very careful and non-trivial analysis of the terms $I^k[i,b]$ defined by :

$$\begin{aligned} I^k[i,b] =\int p^k \times Pr_\pi \big (\langle i\;,b\;\rangle \big )\, dp \end{aligned}$$

with* i*: position along alignment,* b*: alignment suffix that is also π-prefix hitting, over the parameter $k \in \big [|b|_1 \ldots\, \ell -i+|b|\big ]$, and their relationship: this leads to their recurrence formula $I^k[i,b] = I^k[i,b0] + I^{k+1}[i,b1] - I^{k+1}[i,b0]$ computed with the [27] algorithm scheme, using an additional internal loop layer for $k \in [|b|_1 \ldots\, \ell -i+|b|]$, and a *non-obvious ordering of the computed terms on k vs |b|* to remain *DP-tractable*.

Even if the algorithm we propose to compute the Hit Integration (in the next paragraph) has the same *theoretical worst case* complexity, its advantages are twofold:

- We propose a dynamic programming algorithm that is *strictly equivalent* to the one previously proposed for the the Bernoulli model : in fact, both model-dependent algorithms can even pool their most *time-consuming* part. Moreover, the automaton used by the dynamic programming algorithm can be previously minimized: this reduction is *greatly appreciated* when multiple seeds are processed.
- We propose a parameter-free approach for the $p_a$ or $p_b$ parameters: it is therefore possible to compute, on *any interval*, how far a seed is optimal; moreover, we will show that the *dominance* criterion can be applied as a pre-processing step.

The Hit Integration $\int _{p_a}^{p_b} Pr_\pi (p,\ell ) \, dp$ can be rewritten by applying the polynomial formula (1) into:

2 $$\begin{aligned} \int _{p_a}^{p_b}\!Pr_\pi (p,\ell ) \, dp & = \!\!\int _{p_a}^{p_b}\!\sum _{m=0}^{\ell } c_{\pi ,m} \, p^m (1-p)^{\ell -m} dp\nonumber \\ & = \!\!\sum _{m=0}^{\ell } c_{\pi ,m}\int _{p_a}^{p_b}\!p^m (1-p)^{\ell -m} dp \end{aligned}$$

Two interesting features can then be deduced from this trivial rewriting.

First, for any constant integers *u* and *v*, since the integral of the polynomial part $\int _{p_a}^{p_b} p^u (1-p)^{v} \, dp = \Big [ p^{u+1} \sum _{k=0}^{v} {v \atopwithdelims ()k} \frac{(-p)^k}{u+k+1} \Big ]_{p_a}^{p_b}$ can be easily computed (as a larger degree polynomial), the integral of the right part of the formula (2) can be pre-computed independently of the counting coefficients $c_{\pi ,m}$, and thus independently of the seed $\pi$. Thus, only $c_{\pi ,m}$ coefficients characterize the seed $\pi$ for *both* the Bernoulli model *and* the Hit Integration model.

Moreover, we can see that, for $0 \le p_a < p_b \le 1$ and for all $m \in [0\ldots\,\ell ]$, the integral $\int _{p_a}^{p_b} p^m (1-p)^{\ell -m} \, dp$ of the right part of the formula (2) is always positive. Therefore, the *dominance between seeds* also can be directly applied on the $c_{\pi ,m}$ coefficients to select dominant seeds before computing the Hit Integration (for any range $[p_a,p_b]$) by applying the formula (2), thereby saving computation time for the optimal set of seeds.

As a consequence, even if the *optimal* seeds selected from the Bernoulli and the Hit Integration models may have different shapes, all such *optimal* seeds are guaranteed to be *dominant* 4 in the sense of [1]. Note that the dominance of a seed can be computed independently of any parameter *p*, or here, any parameters $[p_a,p_b]$: the dominance criterion can thus be used to pre-select seeds using exactly the same process proposed at the end of the previous part.

**Fig. 4:**
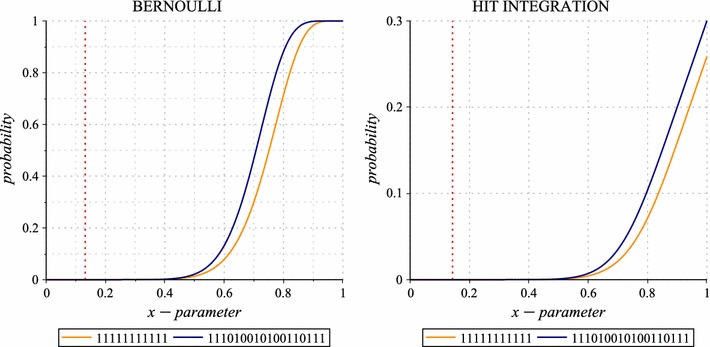
Bernoulli and Hit Integration polynomials. The Bernoulli and $\int _0^x$ Hit Integration polynomials plots for the contiguous seed and the Patternhunter I spaced seed, on alignments of length $\ell =64$. The two polynomials have been plotted according to their respective formulas (1) and (2). A* vertical mark* indicates where they cross each other in the range $x \in \, ]0,1[$ : the contiguous seed is better under this marked value; otherwise, the Patternhunter I spaced seed is better

As an illustration, Fig.  4 plots the Bernoulli (*left*) and the $\int _0^x$ Hit Integration (*right*) polynomials of two seeds: the contiguous seed (11111111111) and the Patternhunter I spaced seed (111010010100110111) which are the two already mentioned out of the forty dominant seeds of weight $w=11$ on alignments of length $\ell =64$. Note that the Patternhunter I spaced seed, when compared to the contiguous seed, turns out to be better, if we consider the Bernoulli criterion only when $p > 0.13209$ (*dark red dashed line*)^5^, or if we consider the $\int _0^x$ Hit Integration criterion only when $x > 0.14301$ (*dark red dashed line*). However, if one wants to consider, not the $\int _0^x$, but the $\int _x^1$ Hit Integration criterion (data not shown), then the Patternhunter I spaced seed will always outperform the contiguous seed, even if both seeds are dominant in terms of $c_{\pi ,m}$ coefficients and cannot be directly compared at first with this *partial order* dominance.

**Fig. 5:**
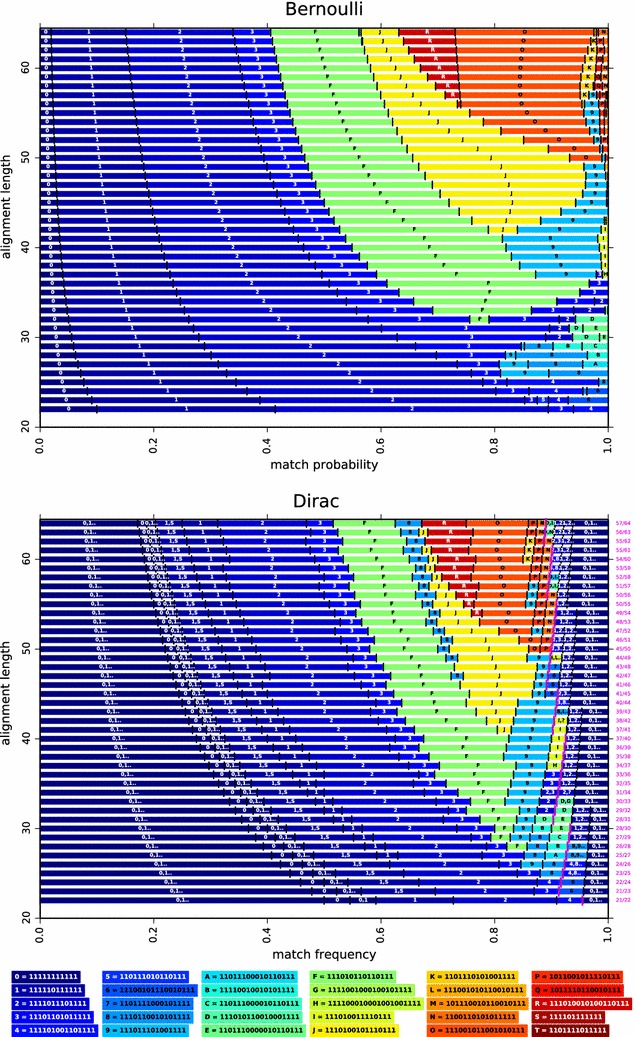
Bernoulli and Dirac optimal seeds. The Bernoulli and Dirac optimal seeds, for single seeds of weight 11 and span $\le 22$, over the match probability or the match frequency of each model (*x*-axis), and on any alignment length $\ell \in [22\ldots64]$ (*y*-axis). On both Figs. 5 and 6, we choose to represent the same seeds with the same label and with the same background color. On discrete models, a pink mark is set. Seeds on the right of this mark are lossless for the two parameters indicated on the right margin: the minimum number of matches *m* over the alignment length $\ell$

**Fig. 6:**
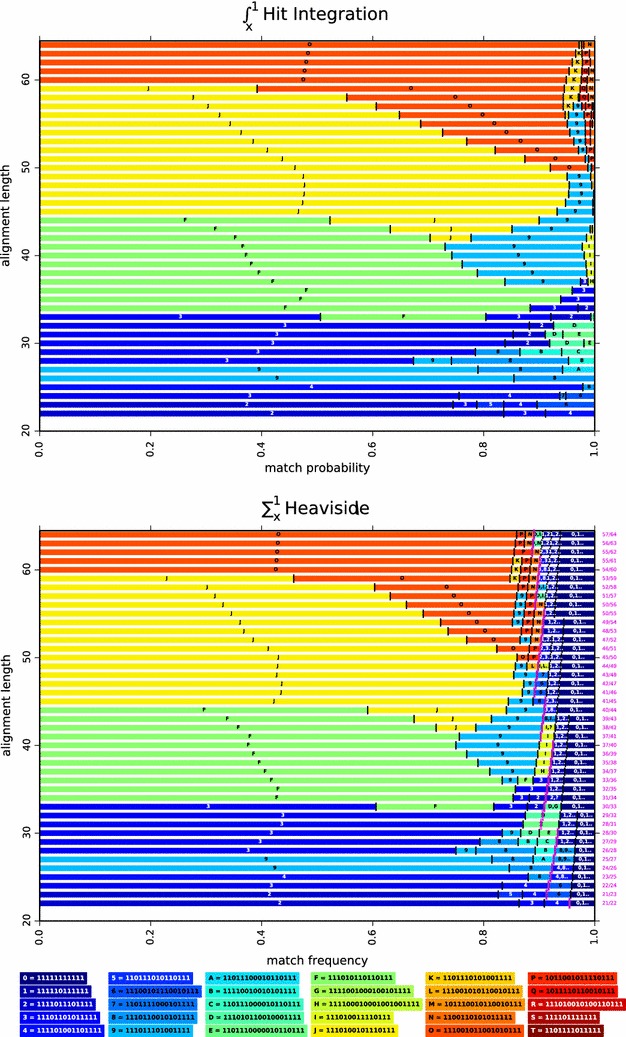
Hit Integration and Heaviside optimal seeds. The $\int _{x}^{1}$ Hit Integration and $\sum _{x}^{1}$ Heaviside optimal seeds, for single seeds of weight 11 and span $\le 22$, over the match probability or the match frequency of each model (*x*-*axis*), and on any alignment length $\ell \in [22\ldots64]$ (*y*-*axis*). On both Figs. 5 and 6, we choose to represent the same seeds with the same label and with the same background color. On discrete models, a* pink mark* is set. Seeds on the right of this mark are lossless for the two parameters indicated on the right margin: the minimum number of matches *m* over the alignment length $\ell$

We finally mention that, for alignments of length $\ell =64$, both the contiguous seed and the Patternhunter I seed are in the set of the twelve optimal seeds found for the Bernoulli model^6^ (they are reported by symbols  and  in Fig.  5,* top line* of the first plot). Both are also in the set of the eight optimal seeds for the $\int _0^x$ Hit Integration model. But, quite surprisingly, neither of the two is in the set of the four optimal seeds for the $\int _x^1$ Hit Integration model (reported in Fig.  6,* top line* of first plot). In fact, for the $\int _x^1$ Hit Integration model, the spaced seed 111001011001010111 (reported by a symbol  in Fig.  6, top line of first plot) is optimal^7^ on a wide range of *x* ($x \in [0,0.97189]$) before being surpassed by three other seeds ( ,  and  in Fig.  6,* top line* of the first plot).

## Discrete models and lossless seeds

In this section, we propose two additional models for selecting seeds. We will name them *Dirac* and *Heaviside*. These models can be seen as the *discrete* counterparts of the Bernoulli and the Hit Integration models, and are simply defined by:

1. $Dirac_\pi (m,\ell ) = \frac{c_{m,\pi }}{{\ell \atopwithdelims ()m}}$, to give the ratio between the number of alignments detected by the seed $\pi$ over all the alignments of length $\ell$ with exactly *m* matches,
2. $Heaviside_\pi (m_a,m_b,\ell ) = \frac{\sum \limits _{m=m_a}^{m_b} Dirac_\pi (m,\ell )}{m_b - m_a + 1}$, to give the average ratio, over any number of matches *m* between $m_a$ and $m_b$ (out of $\ell$) of the previously defined Dirac model. The *Heaviside* full distribution has already been illustrated in Fig.  3, together with the *Hit Integration* distribution with similar parameters.

As long as we allow the possible loss of some of the *strictly equivalent* 8 seeds in terms of sensitivity defined by the Dirac and Heaviside functions, the *dominance* criterion can be applied to filter out many candidate seeds.

In addition, the Dirac and Heaviside functions are based on *rational number* computations/comparisons: they are thus one or two orders of magnitude faster and lighter to compute and store, compared to the polynomial forms given by the continuous models of the previous section.

Finally, an interesting feature of the $Dirac_\pi (m,\ell )$, also true for the specific $Heaviside_\pi (m,\ell ,\ell )$, is that, when the number of match symbols *m* is large enough, one seed $\pi$ (or sometime several seeds) can meet the equality $c_{\pi ,m'} = {\ell \atopwithdelims ()m'}$ for all $m' \ge m$. Such seeds are thus lossless since they can detect all the alignments of length $\ell$ with at least *m* matches (or with at most $\ell -m$ mismatches), and obviously the best lossless ones are retained in the set of dominant seeds, when the equality $c_{\pi ,m} = {\ell \atopwithdelims ()m}$ occurs. As a side consequence, the best *lossless seeds* are also in the set of *dominant seeds* and will be reported in the experiments.

Note that, to keep a symmetric notation with the $\int _{p_a}^{p_b}\,$ *Hit Integration*, and also have the same range for the domain of definition ($0 \le p_a < p_b \le 1$), we will use the “frequency” notation $\sum _{f_a}^{f_b}\,$ *Heaviside* to designate $Heaviside(\lfloor \ell \times f_a \rfloor ,\lfloor \ell \times f_b \rfloor ,\ell )$. We will also rescale the *Dirac* function on the *Bernoulli’s* domain of definition, by using the frequency *f* ($0 \le f \le 1$) to designate $Dirac(\lfloor \ell \times f \rfloor ,\ell )$.

## Experiments

Single spaced seeds ($n =1$) and multiple co-designed spaced seeds ($n \in [2\ldots\,4]$) of weight $w \in [3\ldots\,16]$ and span *s* at most $2 \times w$ have been considered. Note that, for single seeds of large weight ($w \ge 15$), or for multiple seed, the full enumeration is respectively burdensome or intractable, so we prefer to apply the hill-climbing algorithm of Iedera [88]: selected dominant spaced seeds are thus *locally dominant*, since it would be computationally unfeasible to guarantee their overall dominance. All the spaced seeds are evaluated on alignments of length $\ell \in [2 \times w\ldots\,64]$.

The main idea during the evaluation, also used by [1] but only for the single Bernoulli criterion and on a single spaced seed, is to split the computation in two distinct stages:

1. Selecting the *set of dominant seeds* is the first stage: it provides a reduced set of candidate seeds. Note that the dominant selection can be applicable without prior knowledge of the sensitivity criterion being used, provided that this sensitivity criterion is established on *i.i.d sequence* alignments (this last requirement is true for the *Bernoulli*, the *Hit Integration*, the *Dirac*, and the *Heaviside* models).
2. Comparing each of the seeds from the *set of dominant seeds* with a sensitivity criterion is the second stage: it usually depends on *at least* one parameter (for example, for the Bernoulli model: the probability *p* to generate a match) which has different consequences on continuous and discrete models:
  For the *Bernoulli* and the *Hit Integration* continuous models, this implies comparing *p*-parametrized or $[p_a,p_b]$-parametrized polynomials: we follow the idea proposed in [1] for the *Bernoulli* model and also apply it on the *Hit Integration* model where we compute the $\int _0^x$ *HI* and the $\int _x^1$ *HI* respectively. Let us concentrate on the Bernoulli model with a (single) free parameter *p*: For two dominant seeds $\pi _a$ and $\pi _b$ and a given length $\ell$, we compute their respective polynomials $Pr_{\pi _a}(p,\ell )$ and $Pr_{\pi _b}(p,\ell )$ and their difference $Pr_{\pi _a - \pi _b}(p,\ell ) = Pr_{\pi _a}(p,\ell ) - Pr_{\pi _b}(p,\ell )$ (an example of its associated coefficients is illustrated on the third column of Table 1), from which zeros in the range $p \in [0,1]$ are numerically extracted using solvers from maple or maxima. Using the *p*-intervals between these zeros, it is then possible to determine whether $Pr_{\pi _a - \pi _b}(p,\ell )$ is positive or negative, and thus which of the two seeds $\pi _a$ or $\pi _b$ is better according to *p*. Finally, the Pareto envelope (*optimal seeds*) can be extracted from the initial set of dominant seeds.
  For the *Dirac* and the *Heaviside* discrete models, this implies comparing, instead of real-valued polynomials, integer numbers for the Dirac model (and respectively rational numbers for the Heaviside model), which is an easier and lighter process. The** Pareto envelope** can then be easily extracted from these discrete models to select the *optimal seeds* from the set of dominant seeds. We have also extracted the lossless part for the *Dirac* and the $\sum _x^1$ *Heaviside* criteria.

In the aforementioned experiments, we noticed that the size of the *set of dominant seeds* was at most $3359$ (with a median size of 57 and an average size of 303 for all the experiments). To briefly illustrate this point, a list of each maximum size in our experiments is provided on Table 2.

**Table 2:** Maximum size of the set of dominant seeds

	*w*													
*n*	3	4	5	6	7	8	9	10	11	12	13	14	15	16
1	2	7	8	13	15	26	23	32	40	45	46	48	74	84
	(*64*)	(*64*)	(*62*)	(*64*)	(*64*)	(*61*)	(*60*)	(*62*)	(*64*)	(*63*)	(*64*)	(*59*)	(*64*)	(*64*)
2	5	12	35	41	52	99	128	197	231	207	350	320	439	376
	(*64*)	(*63*)	(*63*)	(*61*)	(*64*)	(*64*)	(*60*)	(*62*)	(*61*)	(*59*)	(*63*)	(*64*)	(*64*)	(*41*)
3	6	26	85	84	204	320	391	485	854	932	1103	1449	1508	1812
	(*60*)	(*64*)	(*64*)	(*62*)	(*64*)	(*60*)	(*56*)	(*56*)	(*62*)	(*64*)	(*64*)	(*41*)	(*64*)	(*63*)
4	7	29	124	190	254	535	811	1041	1450	1908	1775	2364	3125	3359
	(*64*)	(*64*)	(*64*)	(*64*)	(*64*)	(*59*)	(*64*)	(*58*)	(*63*)	(*64*)	(*62*)	(*39*)	(*63*)	(*37*)

For *n* seeds of weight *w*, we indicate the maximum size of the dominant set found in our experiments on all the alignment lengths $\ell \in [s\ldots64]$. We also give the largest alignment length $(\ell )$ where this maximum has been reached

So far, we restricted the span of our designed seeds to $2 \times w$, and also did not consider one single fixed probability *p* during the optimization process. These restrictive conditions could be of course alleviated, but we mention here that computed sensitivities are close to (even if not strictly speaking “better than”) the top ones mentioned in several publications [56, 77, 78, 80] where the emphasis was on the heuristic being used for designing seed, the speed of the optimization algorithm, and the best seed for a fixed probability *p*. Table 3 has been extracted from the Table 1 of recently published paper [80] and summarizes known optimal sensitivities.

**Table 3:** Sensitivity comparison of different programs

*w*	*p*	SpEED	AcoSeed	FastHC	MuteHC	Rasbhari	Current sensitivity ($\delta$)
10	0.75	90.9098	90.9513	90.7312	*92.6812*	90.9614	90.8753 (1.8059%)
	0.80	97.8337	97.8521	97.7625	*98.3836*	97.8554	97.8203 (0.5633%)
	0.85	99.7569	99.7614	99.7431	*99.8356*	99.7618	99.7568 (0.0788%)
11	0.75	83.3793	*83.4728*	83.3068	83.4127	83.4679	83.4297 (0.0431%)
	0.80	94.9861	95.037	94.9453	95.0194	*95.0386*	95.0127 (0.0259%)
	0.85	99.2431	99.2478	99.2250	99.2486	*99.2506*	99.2452 (0.0054%)
12	0.80	90.5750	90.6328	90.4735	90.5820	*90.6648*	90.5571 (0.1077%)
	0.85	98.1589	98.1766	98.1199	98.1670	*98.1824*	98.1591 (0.0233%)
	0.90	99.8821	99.8853	99.8771	99.8836	*99.8864*	99.8840 (0.0024%)
16	0.85	84.8212	*84.9829*	84.6558	84.8764	84.969	84.9668 (0.0161%)
	0.90	97.4321	97.4712	97.3556	97.4460	*97.5035*	97.4730 (0.0305%)
	0.95	99.9388	99.9419	99.9347	99.9424	*99.9441*	99.9414 (0.0027%)

Italic values indicate the best sensitivity

The reported sensitivity for $n=4$ seeds of weight *w* on alignments of length $l=50$ under a Bernoulli model with a match probability *p*. All the reported results are extracted from the Table 1 of [80], but the last column that corresponds to our current public seeds, with a $\delta$ difference to the optimal seed

Note that we did not use any *Overlap Complexity*/*Covariance* heuristic optimisation here (to stay in a generic framework), and simply apply the very simple hill-climbing algorithm of Iedera. We also mention that our seeds are not definitely the best ones, but since they are published, their sensitivity can be checked using other software, as mandala [63], SpEED [56], or rasbhari [80] ([43, 57] did the same with the seeds obtained with the SpEED software).

Finally, to show a typical output of this generalized parameter-free approach, optimal single ($n=1$) seeds of weight $w=11$ have been plotted according to the main parameter of each model (horizontal axis) and the length $\ell$ of the alignment (vertical axis) in Figs.  5 and 6. On discrete models, a pink mark represents the lossless border: seeds on the right of this border are by essence **lossless** for the set of parameters. On the right margin of the discrete models, we indicate the fraction of the minimum number of matches *m* over the alignment length $\ell$ to be *lossless*.

We provide the scripts and the whole set of single and multiple seeds, in http://bioinfo.cristal.univ-lille.fr/yass/iedera_dominance in the hope this will be useful to alignment software and spaced seeds alignment-free metagenomic classifiers.

## Discussion

In this paper, we have presented a generalization of the usage of dominant seeds, first on the Hit integration model with a parameter-free approach, and also on two new discrete models (named Dirac and Heaviside) that are related to lossless seeds. In this parameter-free context, we show that all these models can be computed with help of a method for counting alignments of particular classes, themselves represented by regular languages, and a counting semi-ring to perform an efficient set size computation.

We open the discussion with the complementary asymptotic problem, before going to finite but multivariate model extensions.

### Complementary asymptotic problem

So far, we only have considered a set of finite alignment lengths $\ell$ to design seeds. *But * limiting the length is far from satisfactory, so the next problem deserves consideration too: the asymptotic hit probability of seeds [63, 89–91].

As an example, if we consider the Bernoulli model where we choose *p* in the interval ]0, 1[, and then consider the probability $Pr_\pi (p,\ell )$ for $\pi$ to hit an alignment of length $\ell$ (noted $Pr_\pi (\ell )$ to simplify), then it can be shown that the complementary probability $\overline{Pr_\pi (\ell )}$ [see for example 91, equation (3)] follows

$$\begin{aligned} \lim _{\ell \rightarrow \infty } \overline{Pr_\pi (\ell )} = \beta _\pi \lambda _\pi ^\ell \big (1+o(1)\big ) \end{aligned}$$

Here $\lambda _\pi$ is the largest (positive) eigenvalue of the sub-stochastic matrix of $\pi$ where final states have been removed, this matrix computing thus the distribution $\overline{Pr_\pi (\ell )}$ when powered to $\ell$ (see section 3.1 $\lambda _\pi$ and $\beta _\pi$ of [63]).

As an example, for $p = 0.7$ and for the Patternhunter I spaced seed, we have (with help of a Maple script) $\{\lambda ,\beta \}_\mathtt{111010010100110111} = \{0.98731,0.22667\}$, that can be compared with the contiguous seed of same weight $\{\lambda ,\beta \}_\mathtt{11111111111} = \{0.99364,0.44784\}$. [63] have proven that, in the class of seeds with the same weight, contiguous seeds have the largest value $\lambda$ and thus are the asymptotic worst-case in terms of hit probability, a trait shared with the *uniformly spaced* seeds of same weight (e.g. 101010101010101010101 or 1001001001001001001001001001001).

Comparing seeds asymptotically can thus be done easily by comparing their respective $\lambda$ eigenvalue, or their $\beta$ when $\lambda$ equality occurs, but it seems to be *computationally possible* 9 only if *p* is set numerically before the analysis.

Moreover *dominant seeds’* extracted from this paper on a limited alignment length $\ell$ (here $\ell \le 64$) would not always be optimal for any $\ell$: such seeds can, however, be justified as “good” candidates for seeds of restricted span (e.g. $s \le 2\times w$), but definitely not the optimal ones, unless dominance is computed on a wider range of alignment length $\ell$ values.

For example, the best (smallest) $\lambda$ for any *dominant* seed of weight $w=11$ and span at most $2 \times w$, on alignments of length $\ell \le 64$ is 0.98714 for the seed 1110010100110010111. Surprisingly, even if this seed reaches the smallest $\lambda$ out of its *dominant* class, it never occurred in the *optimal* seeds, in any of our experiments. Moreover, we have checked that another seed 1110010100100100010111 has an even smaller $\lambda = 0.98669$: this last seed was not dominant for $\ell \le 64$, but would be in the class of seeds of span at most $2 \times w$ if larger values of $\ell$ were selected.

Finally, a parameter-free analysis implying both *p* and $\ell$ seems difficult to apply for large seeds. It is interesting to notice that several of our preliminary experiments *suggest* that, asymptotically, and only^10^ for a *restricted set* of seeds (e.g. of weight $w=11$ and span at most $2 \times w$), *one seed is optimal whatever the value of p*. This remains to be confirmed experimentally and theoretically because it might be possible that special cases exist, where at least two (or even more) seeds share the *p* partition.

### Models and multivariate analysis

As far as *i.i.d sequences* are considered, the full framework of [1], including the dominant seed selection, can be applied on *any extended spaced seed model* (such as transition constrained seeds, vector seeds, indel seeds,...). However, additional free-parameters (such as the transition/transversion rate, the indel/mismatch rate, ...) lead to an increase in the number of alignment classes (for example, alignments of length $\ell$, with *i* indels, *v* transversion errors, *t* transitions errors, and remaining *m* matches, such that $\ell =i+v+t+m$) that have to be considered by the dominance selection. Moreover, it involves a much more complex multivariate polynomial analysis, if more than one parameter is, at this point, left free.

In a more general way, if *i.i.d sequences* are ignored, and dominant seed selection thus abandoned in its original form, one could mix several numerically-fixed models: for example, mixing a given HMM representing coding sequences, with a numerically-fixed Bernoulli model. The idea is here to use a *free probability parameter* to create a balance between the two models: either initially before generating the alignment, to choose each of the two models; or along the alignment generation process, to switch between each of the two models. Seeds designed could thus be *two-handed* for analyzing both coding and non-coding genomic sequences at the same time, but with an additional control parameter that helps to change the known percentage of such genomic sequences. To compute the sensitivity in this model, a simple idea is to apply a polynomial semi-ring (with at least one parameter-free variable: here the one used to create the balance) on the automaton, and perform, not a numeric, but a symbolic computation.

Finally, as a logical consequence of the two previous remarks, we mention that any HMM with one (or possibly several) free probability parameter(s) could always be analysed with a (multivariate) polynomial semi-ring, increasing thus the scope of the method to applications that depend on Finite State Machines : such parameter-free pre-processing can, at some point, be applied; moreover if several equivalence classes are established in term of probability, it may be possible to use equivalent dominance method to filter out candidates when comparing several elements.

## Footnotes

1 We mention an interesting analysis in [92].

2 The opposite *is equivalent* to say that *at least one* string of length $\ell$ with $\le k$ mismatches is not hit by the seed; in other words, that the seed is not $(\ell ,k)$-lossless. Note that *k* does not need to be initially set: it can be estimated using this requirement, even after the DP run.

3 Technical details at http://bioinfo.cristal.univ-lille.fr/yass/iedera_coverage/index_additional.html.

4 This side result is not discussed in [2], probably because they were more interested by the seed rank and not necessary the “optimal seed”, which they sometime called “dominant”.

5 As already observed by [63].

6 As already mentioned by [1].

7 As already mentioned by [2], but for the non-parametrized $\int _0^1$ and $\int _{\frac{1}{2}}^1$ Hit Integration model.

8 To give a quick and intuitive example, we consider an extreme case : an alignment of fixed length $\ell$ without any mismatch symbol. Any seed $\pi$ of weight $w_\pi \le \ell$ and span $s_\pi \le \ell$ obviously detects this alignment, whatever its shape is, so $Dirac_\pi (m=\ell ,\ell )$ and $Heaviside_\pi (m_a=\ell ,m_b=\ell ,\ell )$ reach their maximal sensitivity of 1. For a given weight *w*, the restriction of all these seeds to dominant seeds implies that many are lost when dominance selection is applied to keep the best representatives.

9 At least to the author, but this parametrized problem is intrinsically interesting in itself.

10 This restricted set of seeds condition is necessary: if removed, best seeds span will increase along $\ell$, see [18].
